# Constipation

**Published:** 1878-05

**Authors:** 


Constipation.—Dr. J. Lewis Smith (Virginia *Medical Monthly*) says that the following pill may be used in those cases not relieved by the use of fruits and laxative articles of diet:

℞.—Ext. belladonnæ, . . . . . gr. iij.
Ext. nucis vomicæ, . . . . . gr. vi.
Podophyllin, . . . . . . gr. vi–ix:
M. Div. in pil. No. xviii.
S. Take one when required.

